# Engineering a TGEV S-trimer chimera with PEDV D0-NTD generates potent neutralizing antibodies against both viruses

**DOI:** 10.1128/jvi.01452-25

**Published:** 2025-10-31

**Authors:** Ding Zhang, Yang Yuan, Guangli Hu, Yunfei Xie, Xiumei Meng, ZhaoTian Zhang, Yu Zhang, Qi Liao, Ashenafi Assefa Gebremariam, Hanqin Shen, Guiqing Peng, Yuejun Shi

**Affiliations:** 1 State Key Laboratory of Agricultural Microbiology, College of Veterinary Medicine, Huazhong Agricultural University 627716, Wuhan, China; 2 Key Laboratory of Preventive Veterinary Medicine in Hubei Province, The Cooperative Innovation Center for Sustainable Pig Production, Huazhong Agricultural University, Wuhan, China; 3 State Key Laboratory of Biocontrol, School of Life Sciences, Sun Yat-sen University 200651 , Guangzhou, China; 4 Guangdong Provincial Enterprise Key Laboratory of Healthy Animal Husbandry and Environment Control, Wen’s Foodstuff Group Co. Ltd 656319 , Yunfu, China; 5 Frontier Science Center for Animal Breeding and Healthy Farming, Huazhong Agricultural University 47895, Wuhan, China; Loyola University Chicago - Health Sciences Campus, Maywood, Illinois, USA

**Keywords:** porcine epidemic diarrhea virus (PEDV), transmissible gastroenteritis virus (TGEV), chimeric spike protein, subunit vaccine, cross-protection, immunogenicity

## Abstract

Porcine epidemic diarrhea virus (PEDV) and transmissible gastroenteritis virus (TGEV), two economically devastating swine enteric coronaviruses, persistently threaten global pork production via severe clinical morbidity. Vaccination is the most effective means to prevent morbidity and mortality caused by porcine enteric coronavirus infection. Hence, the development of an effective and safe vaccination strategy to protect against both PEDV and TGEV infections is urgently needed. Here, we designed and produced trimerized full-length PEDV and TGEV spike (S) proteins using an efficient mammalian expression vector system in HEK293F cells and reported that compared with the PEDV spike protein (2^7^), the TGEV spike protein induces significantly more potent neutralizing antibodies (2^12^). Moreover, transmission electron microscopy (TEM) with negative staining revealed that the TGEV spike protein exhibited excellent homogeneity. We subsequently used the TGEV S protein trimer as the backbone to display different domains of the PEDV S protein and successfully constructed seven spike protein chimeras. We further screened two chimeric S proteins through expression and mouse experiments, which included TGEV S-PEDV(D0/NTD) and TGEV S-PEDV(D0/NTD/CTD), in which the S protein of TGEV was replaced by the PEDV S D0-NTD and D0-NTD-CTD, respectively. Evaluation of the immunogenicity and efficacy of the chimeric S proteins in piglets confirmed that piglets in the TGEV S-PEDV(D0/NTD)-immunized group generated high levels of neutralizing antibodies (2^13.6^ against TGEV; 2^5.8^ against PEDV) against both PEDV and TGEV. Our findings suggest that the TGEV S-PEDV(D0/NTD) is a promising candidate for combating both PEDV and TGEV infections.

**IMPORTANCE:**

The design strategy of multivalent and multitarget single antigens facilitates the development of vaccines targeting PEDV and TGEV. Optimizing the presentation of PEDV core antigen epitopes on the basis of the S protein trimer structure will provide novel insights into the development of subunit vaccines. Here, we compared the immunogenicities of the TGEV S protein and PEDV S protein and then designed a bivalent chimeric S protein candidate, TGEV S-PEDV(D0/NTD), in which the corresponding segments on the TGEV S protein were replaced with D0-NTD domains from the PEDV S protein. We demonstrated that the TGEV S protein exhibits greater immunogenicity than the PEDV S protein and that TGEV S-PEDV(D0/NTD) induces broad-spectrum neutralization protection against PEDV and TGEV. Our results demonstrate the effectiveness of the chimeric S protein and provide a feasible method for the development of efficient bivalent subunit vaccines against PEDV and TGEV.

## INTRODUCTION

Porcine epidemic diarrhea (PED) and transmissible gastroenteritis (TGE), caused by porcine epidemic diarrhea virus (PEDV) and transmissible gastroenteritis virus (TGEV), respectively, are two highly contagious intestinal diseases affecting the global swine industry (1–6). All ages of swine are highly susceptible to PEDV and TGEV infection, particularly piglets within 2 weeks of age, which show a notably high mortality rate (7, 8). Before 2010, PEDV infections in China caused sporadic and endemic outbreaks. Since October 2010, a severe epidemic caused by the highly pathogenic porcine epidemic diarrhea virus (PEDV) has been continuously spreading among pig populations in China, resulting in considerable economic losses (6, 9–12). TGEV was first described in the United States in 1946 and was subsequently found in Europe, Asia, Africa, and South America, causing major losses to the global pig farm industry (13–16). The clinical symptoms caused by TGEV are similar to those caused by PEDV, and they often present clinically mixed infections. However, cross-protection between the two viruses is limited (17). Therefore, there is an urgent need to develop a novel protective, safe, and affordable vaccine against both PEDV and TGEV.

Both PEDV and TGEV are members of the *Alphacoronavirus* genus (18, 19), and the genomes of PEDV and TGEV are approximately 28 kb in length with 5′-capped and 3′-polyadenylated untranslated regions (UTRs) (20, 21). The genomes of both PEDV and TGEV are conventionally organized around seven major open reading frames. These include the replicase genes ORF1a and ORF1b, encoding the pp1a/pp1ab polyproteins that are processed into 16 nsps (1–16) for replication and transcription, and the genes for the structural proteins Spike (S), Envelope (E), Membrane (M), and Nucleocapsid (N) and the ORF3 gene encoding an accessory protein (20). Among these viral proteins, the S protein on the surface of the virus is the main envelope glycoprotein. The trimeric structures of the PEDV S protein have been successively determined (22–24). The S protein comprises S1, a receptor-binding subunit that is responsible for host cell attachment and receptor binding, and S2, a membrane fusion subunit that is involved in triggering the fusion of the viral envelope and target cell membrane during infection (25, 26). The S1 region (cell attachment domain) is considered a key target of neutralizing antibodies, and the S1-D0 and NTD regions induce the production of additional neutralizing antibodies (27). Therefore, the S1-D0 and S1-NTD regions may be key epitopes of the PEDV S protein. Currently, PEDV strains are classified into genotypes G1 and G2, with the G2 genotype being the clinically prevalent strain. The G2 genotype is further divided into the G2a, G2b, and G2c strains, among which the G2c strain has replaced G2a and G2b to become the dominant prevalent strain (10, 28, 29). Faced with the rapid evolution of PEDV genotypes and immune evasion, monitoring the variation and evolution of coronaviruses like PEDV and developing corresponding vaccines have become a critical priority in the clinical prevention and control of this virus (30). Vaccination is one of the most important measures for controlling PEDV and TGEV outbreaks (20, 31). Recently, researchers have developed a PEDV-TGEV-PDCoV trivalent inactivated vaccine, and it has been shown to provide effective protection in pigs against all three enteric coronaviruses (32). Both inactivated and live-attenuated virus vaccines are commercially available and have been widely used to prevent PEDV and TGEV infection (33, 34). However, the emergence of highly virulent strains and recurrent outbreaks, even on vaccinated farms, highlights the limitations of traditional vaccines and the need for effective vaccines (35). Compared with total viral vaccines, protein vaccines of subunits have several advantages that make them appealing for the development of new vaccines, such as enhanced safety features, elimination of infectious viral genetic material, antigen structural redesign, multi-antigen combinations, and strategic adjuvant integration; this approach significantly improves therapeutic efficacy (36, 37). Therefore, our primary focus is to develop a chimeric S protein subunit vaccine against both PEDV and TGEV.

PEDV and TGEV are two major coronaviruses responsible for severe diarrhea and mortality in piglets. Because of the rapid mutation of PEDV, methods to quickly develop a vaccine for the effective prevention and control of both viruses are urgently needed. Here, we designed seven chimeric S proteins encoding different domains of the PEDV S1 region with the TGEV S protein trimer as the backbone. We investigated the expression levels, immunogenicity, and protective efficacy of these chimeric S proteins *in vitro* and in mice. Piglets were then immunized with two chimeric S proteins [TGEV S-PEDV(D0/NTD) and TGEV S-PEDV(D0/NTD/CTD)] to evaluate their broad-spectrum immunogenicity against PEDV and TGEV. Our results demonstrated that the TGEV S-PEDV(D0/NTD) induced better immunity than the TGEV S-PEDV(D0/NTD/CTD) did, suggesting a promising strategy for preventing PEDV and TGEV infections.

## RESULTS AND DISCUSSION

### Expression and purification of recombinant proteins

**Fig 1:**
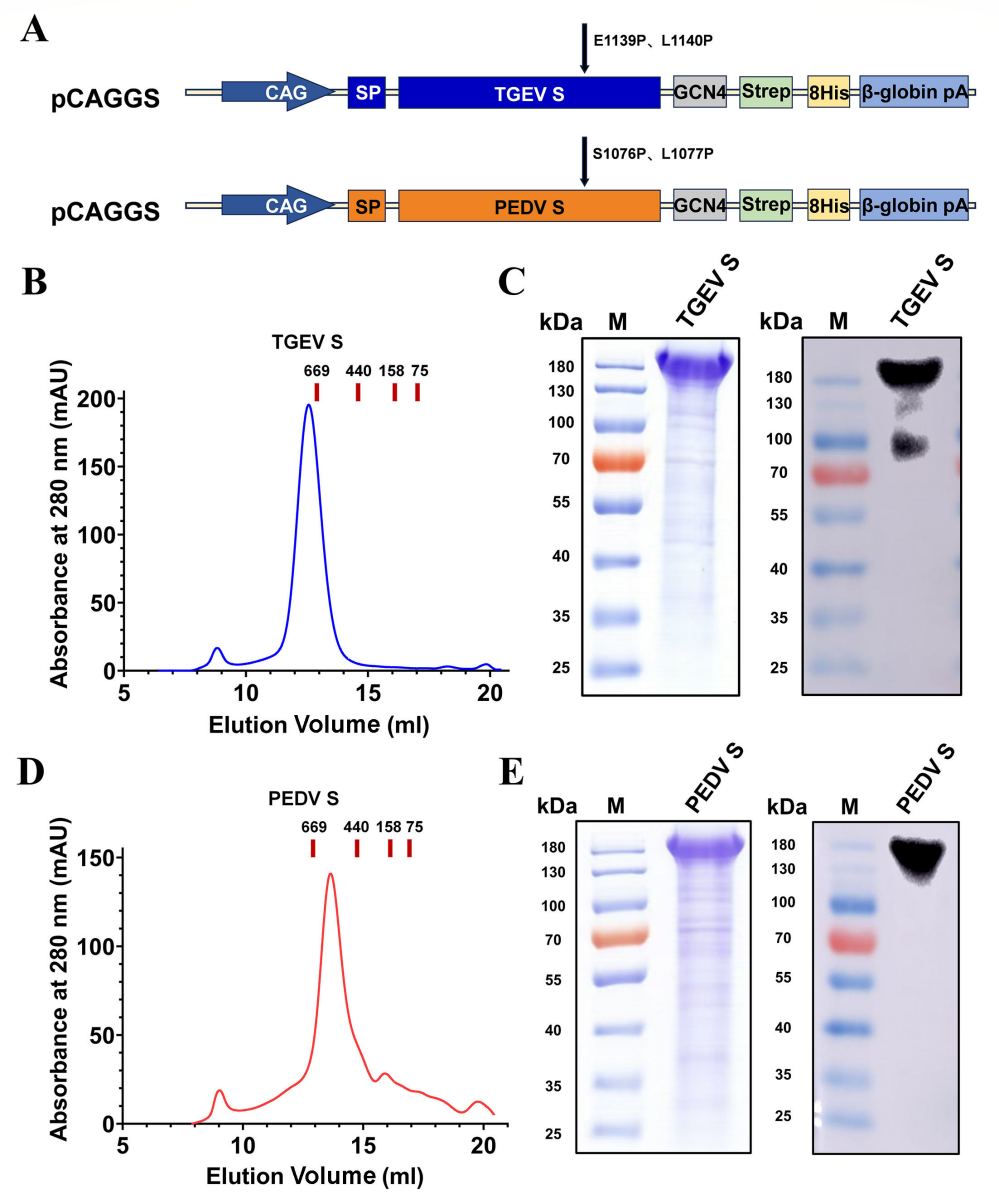
Expression and purification of the TGEV and PEDV S-trimer. (**A**) Schematic diagram of the construction of the trimeric TGEV S protein and PEDV S protein. (**B**) SEC‒HPLC analysis of purified TGEV S protein. (**C**) Analysis of TGEV S protein by SDS‒PAGE (left panel) and western blotting (right panel). (**D**) SEC‒HPLC analysis of purified PEDV S protein. (**E**) Analysis of PEDV S protein by SDS‒PAGE (left panel) and western blotting (right panel).

To increase antigen expression, the TGEV S and PEDV S genes were optimized and synthesized, and we engineered TGEV and PEDV spike trimers by incorporating the GCN4 trimer domain, a Strep-tag II, and an 8× His tag. For each, we introduced the 2P mutation (E1139P/L1140P for TGEV S; S1076P/L1077P for PEDV S) and removed 59 or 67 residues from the C-terminus, respectively. As shown in Fig. 1A, the cDNA encoding the recombinant proteins was subcloned and inserted into the pCAGGS expression vector and successfully expressed in HEK293F cells. Proteins were purified by His-tag affinity chromatography, followed by size exclusion chromatography (SEC). The SEC chromatogram of the purified proteins revealed that TGEV S-Trimer eluted as a single peak with a molecular weight corresponding to a molecular weight of above 669  kDa and that PEDV S-Trimer eluted as a single peak with a molecular weight at or below approximately 669 kDa, suggesting that both TGEV S-Trimer and PEDV S-Trimer predominantly form a heavily glycosylated homotrimer (Fig. 1B and D). The expression and purification of the TGEV S protein and the PEDV S protein were confirmed through SDS‒PAGE and western blot analyses. As shown in Fig. 1C and E, the bands corresponding to TGEV S-monomer and PEDV S-monomer appeared near the protein standard at 180 kDa, consistent with the molecular weight of a glycosylated monomer that showed no evidence of cleavage by endogenous proteases. These results indicated that purified trimeric TGEV S and PEDV S antigens were obtained.

### The ability of the TGEV S protein to induce neutralizing antibodies is superior to that of PEDV

**Fig 2:**
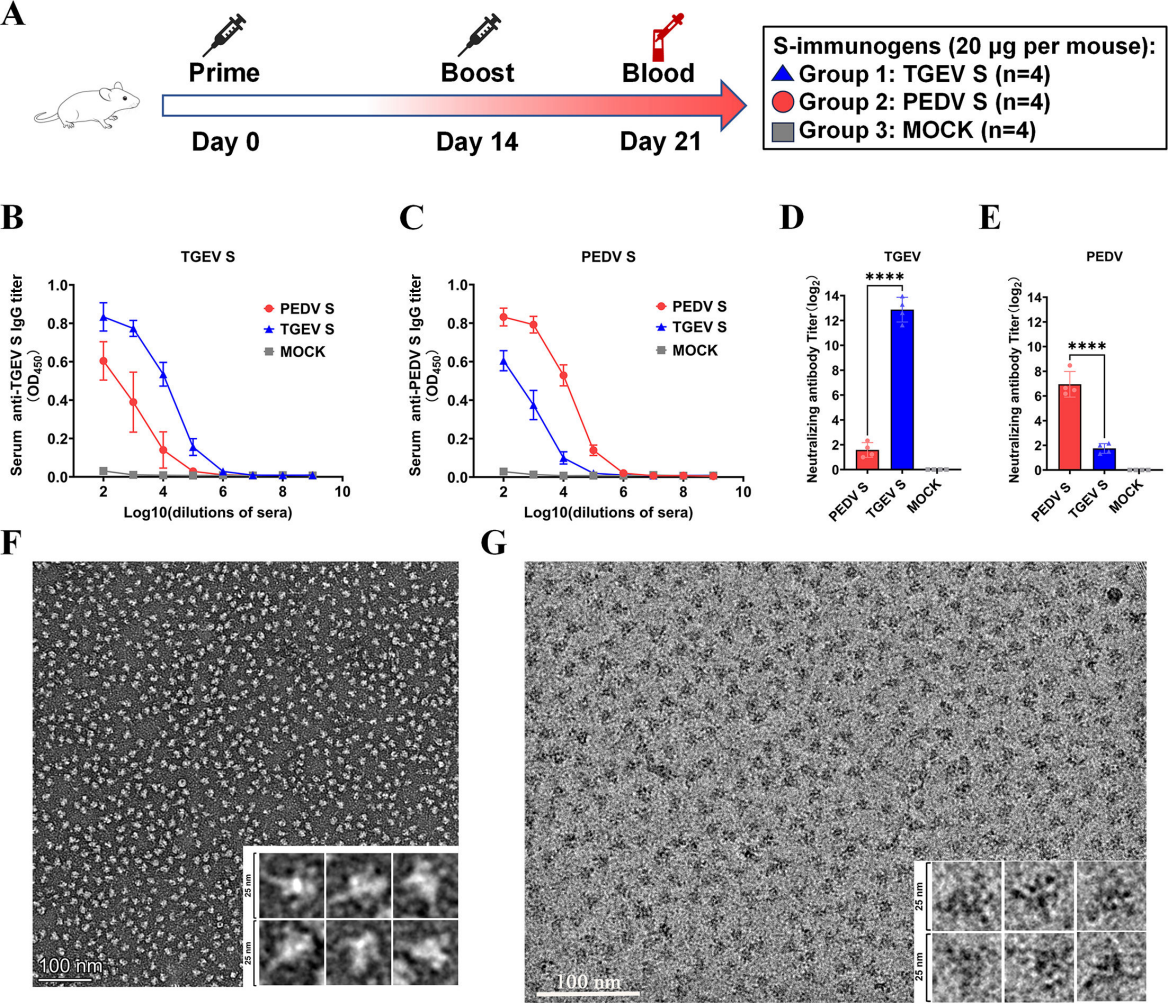
Evaluation of the immunogenicity of TGEV S and PEDV S in mice. (**A**) Experimental design for mouse vaccination. (**B and C**) Serum levels of TGEV S (**B**) and PEDV S (**C**) protein-binding IgG antibodies were measured at 21 d by ELISA. (**D and E**) Serum-neutralizing antibodies against TGEV (**D**) and PEDV (**E**) were detected. (**F**) An example negative-stain micrograph of TGEV S protein. (**G**) An example cryo-EM micrograph of TGEV S protein. The data are presented as the means ± SDs of four mice per group. The asterisks in the figures indicate significant differences (****P < 0.0001).

We next compared the immunogenicity of TGEV S with that of PEDV S in a mouse model. The study design and experimental groups are summarized in Fig. 2A: group 1 included mice inoculated with TGEV S (*n* = 4); group 2 included mice inoculated with PEDV S (*n* = 4); and group 3 included mice inoculated with normal saline as a mock (*n* = 4). All the mice in groups 1 and 2 received two inoculations at 2‐week intervals. Blood was collected from the mice on day 21. The mice experienced no adverse events after vaccination. Serum samples were collected to evaluate antibody responses using enzyme-linked immunosorbent assay (ELISA) or virus neutralization tests. As shown in Fig. 2B and C, the TGEV S-specific IgG titer response to the TGEV S protein was similar to the PEDV S-specific IgG titer response to the PEDV S protein. However, at the specific IgG antibody level, immunization with TGEV S resulted in a weaker cross-reaction than with PEDV S, and vice versa.

In addition to the IgG response, the neutralizing ability of antibody production is a pivotal factor influencing the quality of immunity. To further evaluate the humoral response induced by S proteins in experimental animals, neutralizing antibodies against TGEV and PEDV were also detected. The levels of TGEV-neutralizing antibodies in the TGEV S-immunized group were significantly higher, with titers of approximately 2^12^ in the serum, than those in the mock group (Fig. 2D). The levels of PEDV-neutralizing antibodies in the PEDV S-immunized group were significantly higher, with titers of approximately 2^7^ in the serum, than those in the mock group (Fig. 2E). The mean TGEV-neutralizing antibody titer in the TGEV S-immunized group was significantly higher than the mean PEDV-neutralizing antibody titer in the PEDV S-immunized group. Moreover, mice immunized with TGEV S and those immunized with PEDV S did not exhibit cross-protection in terms of serum neutralizing antibodies (Fig. 2D and E). The results from transmission electron microscopy (TEM) analysis of negatively stained samples (120 kV) and cryo-EM samples (300 kV) demonstrated high homogeneity of the TGEV spike protein (Fig. 2F and G), with the vast majority of particles exhibiting well-defined triangular morphology, demonstrating exceptional structural homogeneity. This well-preserved native-like architecture likely contributes to the robust immunogenicity of the TGEV S protein. Our results indicate that the TGEV S elicits a stronger humoral immune response than the PEDV S in mice.

### Antigenic epitopes of TGEV and PEDV S proteins are primarily located in the S1-D0, S1-NTD, and S1-CTD regions

**Fig 3:**
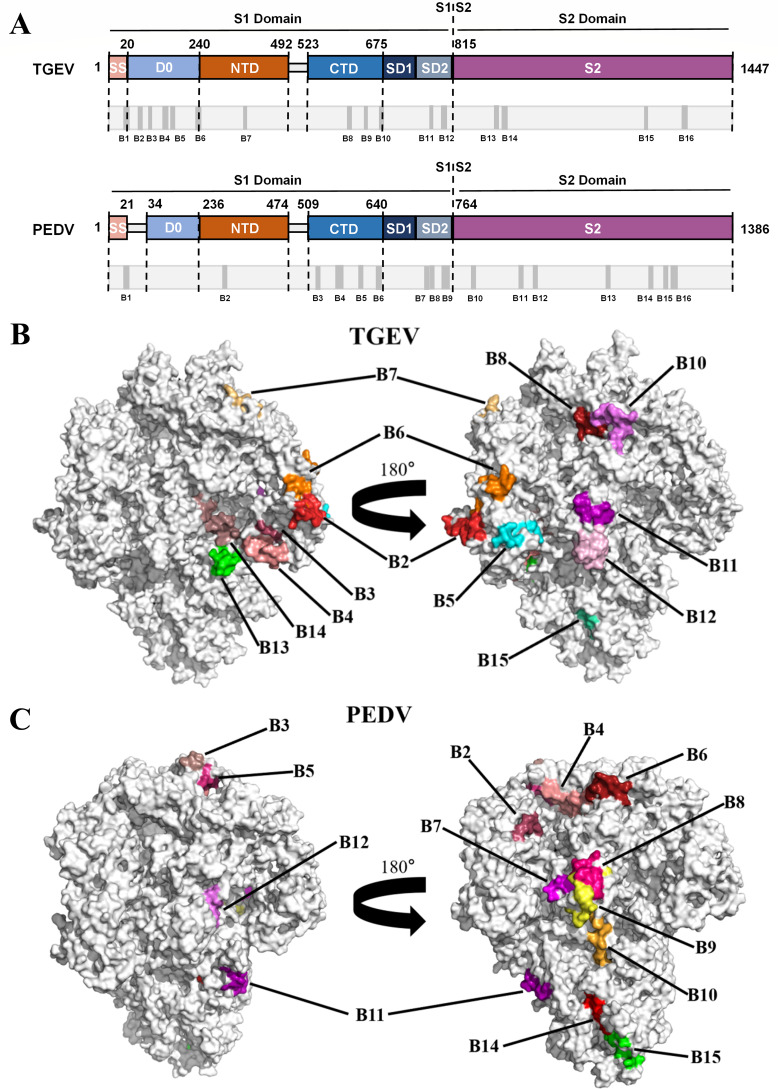
B-cell linear epitopes of the TGEV S protein and PEDV S protein screened by bioinformatics. (**A**) The primary structures of the TGEV S (upper panel) and PEDV S (lower panel) proteins were analyzed, and the precise locations of the B-cell linear epitopes within the S proteins were elucidated. (**B and C**) Surface representation of the B-cell linear epitopes on the 3D structure of the trimeric TGEV S (**B**) and PEDV S proteins (**C**). These structures were predicted by the SWISS-MODEL server (http://swissmodel.expasy.org/) using the S protein of PEDV as a template (PDB ID: 7W6M).

**Fig 4:**
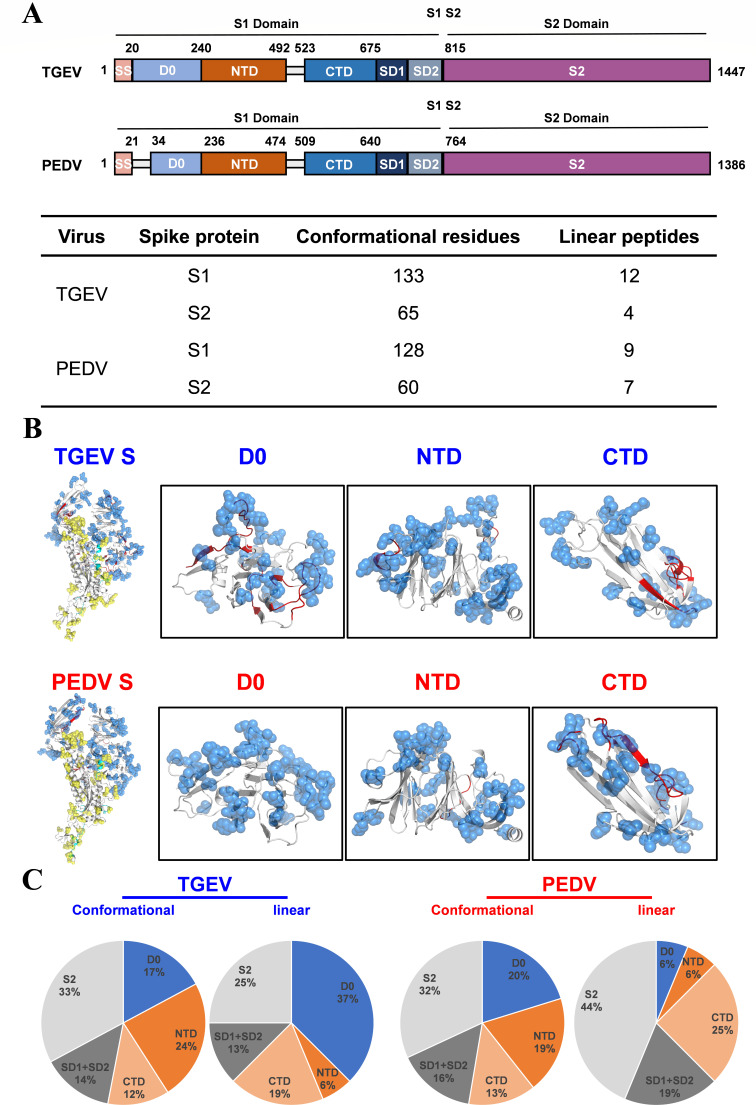
Structure-based B-cell epitope predictions of TGEV S and PEDV S. (**A**) Schematic diagram of TGEV S and PEDV S. S1, receptor-binding subunit; S2, membrane fusion subunit; SS, signal sequence; D0, domain 0; NTD, N-terminal domain of S1; CTD, C-terminal domain of S1; SD1, subdomain 1 of S1; SD2, subdomain 2 of S1. The linear and conformational epitopes of B cells on S1 and S2 of TGEV S and PEDV S are summarized. (**B**) The structure-based predicted B-cell epitopes of TGEV S and PEDV S are shown. The linear (red cartoon for S1, cyan cartoon for S2) and conformational (marine blue sphere for S1, yellow blue sphere for S2) B-cell epitopes were predicted and labeled in the corresponding structure via PyMOL. (**C**) The ratio of B-cell epitopes in each structural domain of the TGEV S protein and the PEDV S protein.

We performed B-cell epitope prediction to analyze the effective linear epitopes of the TGEV S protein and the PEDV S protein. A total of 16 B-cell linear epitopes were identified in TGEV S and PEDV S (Fig. 3A). The predicted positive residues of TGEV S and PEDV S (the corresponding linear epitopes) are displayed on the structural surface (Fig. 3B and C). Among these epitopes, 12 and 9 B-cell linear epitopes were located in the S1 domain of TGEV and PEDV, respectively (Fig. 4A). The results show that B-cell linear epitopes in both proteins were predominantly located in the S1 region.

To further determine which subdomains within the S1 subunit harbor the highest density of B-cell epitopes, structural and discontinuous epitopes in the TGEV S and PEDV S proteins were predicted with the DiscoTope 3.0 server. A total of 198 and 188 amino acid residues located on the S protein were predicted to be conformational epitopes for TGEV and PEDV, respectively. Among these residues, 133 and 128 residues were located in the S1 domain of TGEV and PEDV, respectively (Fig. 4A). The above findings demonstrate that the number of predicted B-cell epitopes on S1 is significantly higher than that on S2 for both TGEV and PEDV. Additionally, the epitopes of TGEV S and PEDV S are contained primarily within the D0, NTD, and CTD subdomains of the S1 subunit (Fig. 4B and C). These results suggest that retaining the different subdomains of S1 with the majority of B-cell epitopes that can induce protective Abs is vital for designing effective chimeric S protein subunit vaccines.

### Optimized expression strategy for the chimeric protein TGEV S-PEDV(D0/NTD)

**Fig 5:**
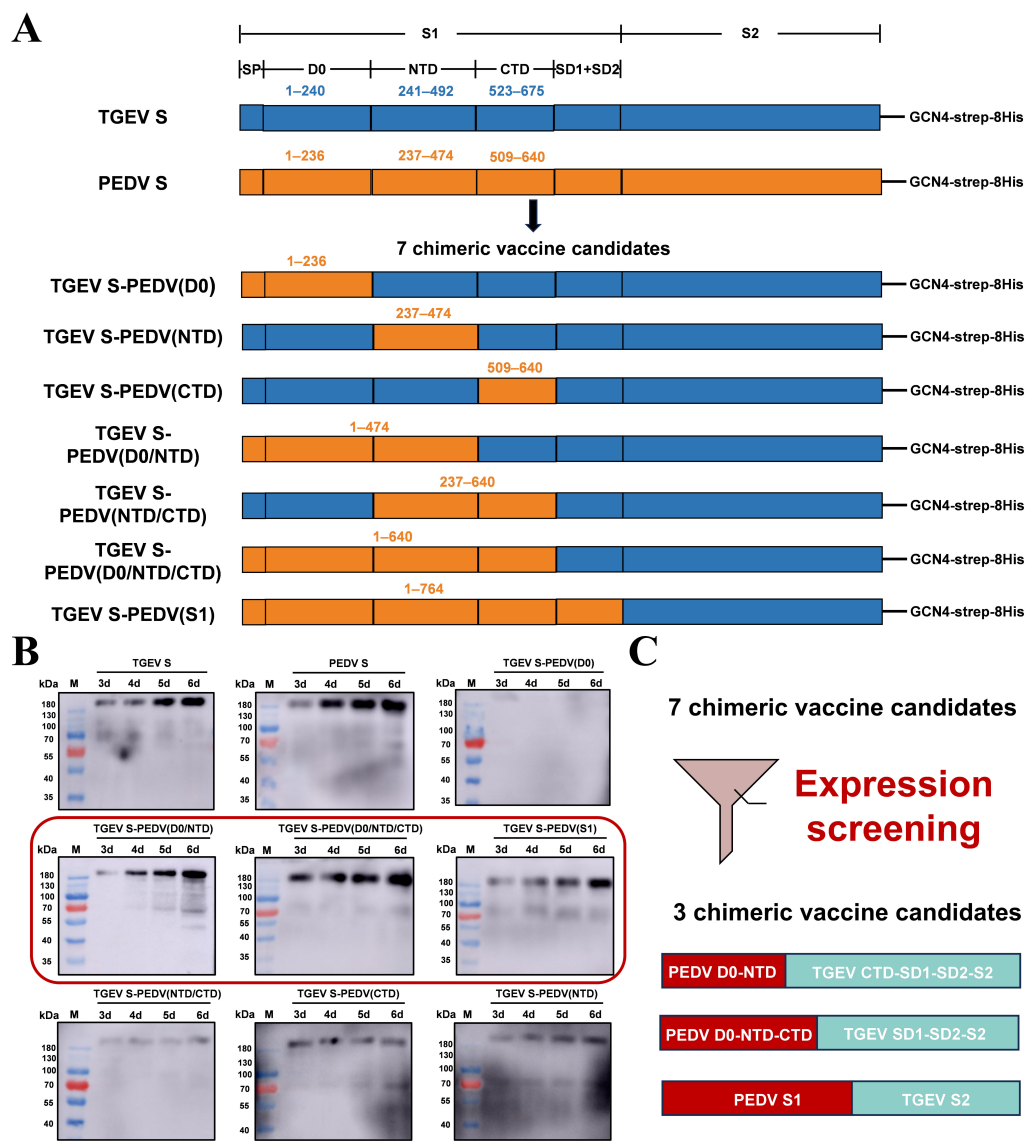
Design and screening of bivalent chimeric S protein candidates. (**A**) Schematic illustration showing the construction of chimeric S proteins. The blue and orange regions represent TGEV S and PEDV S, respectively. (**B**) Western blot showing different expression levels of chimeric S proteins in the cell supernatant from day 3 to day 6. (**C**) Schematic diagram of the screening results for chimeric S protein candidates.

Our previous study suggested that in alpha-CoVs, subunit vaccines should prioritize the S-trimer rather than the S1-RBD (38). Moreover, the above results demonstrate that the immunogenic efficacy of the TGEV S protein surpasses that of the PEDV S protein, and the TGEV spike protein exhibited excellent homogeneity. Therefore, we engineered seven chimeric spike proteins with the TGEV S protein trimer as the backbone, which expressed different domains of the PEDV S1 subunit (Fig. 5A). To preliminarily screen suitable chimeric S proteins, the expression levels of these S protein chimeras on different days were determined by western blot analysis. As shown in Fig. 5B, among the seven chimeric spike proteins, the expression levels of three spike protein chimeras—TGEV S-PEDV(D0/NTD), TGEV S-PEDV(D0/NTD/CTD), and TGEV S-PEDV(S1)—were close to those of TGEV S and PEDV S, whereas the other four chimeras presented lower expression levels. In these constructs, the D0-NTD domain (aa 1–492) within the TGEV spike trimer was replaced with the corresponding PEDV D0-NTD region (aa 1–474) [designated TGEV S-PEDV(D0/NTD)], the D0-NTD-CTD domain (aa 1–675) within the TGEV spike trimer was replaced with the corresponding PEDV D0-NTD-CTD region (aa 1–640) [designated TGEV S-PEDV(D0/NTD/CTD)], and the S1 domain (aa 1–815) within the TGEV spike trimer was replaced with the corresponding PEDV S1 region (aa 1–764) [designated TGEV S-PEDV(S1)]. The results indicated that we successfully screened three chimeras with high expression levels as candidate chimeric S proteins (Fig. 5C).

**Fig 6:**
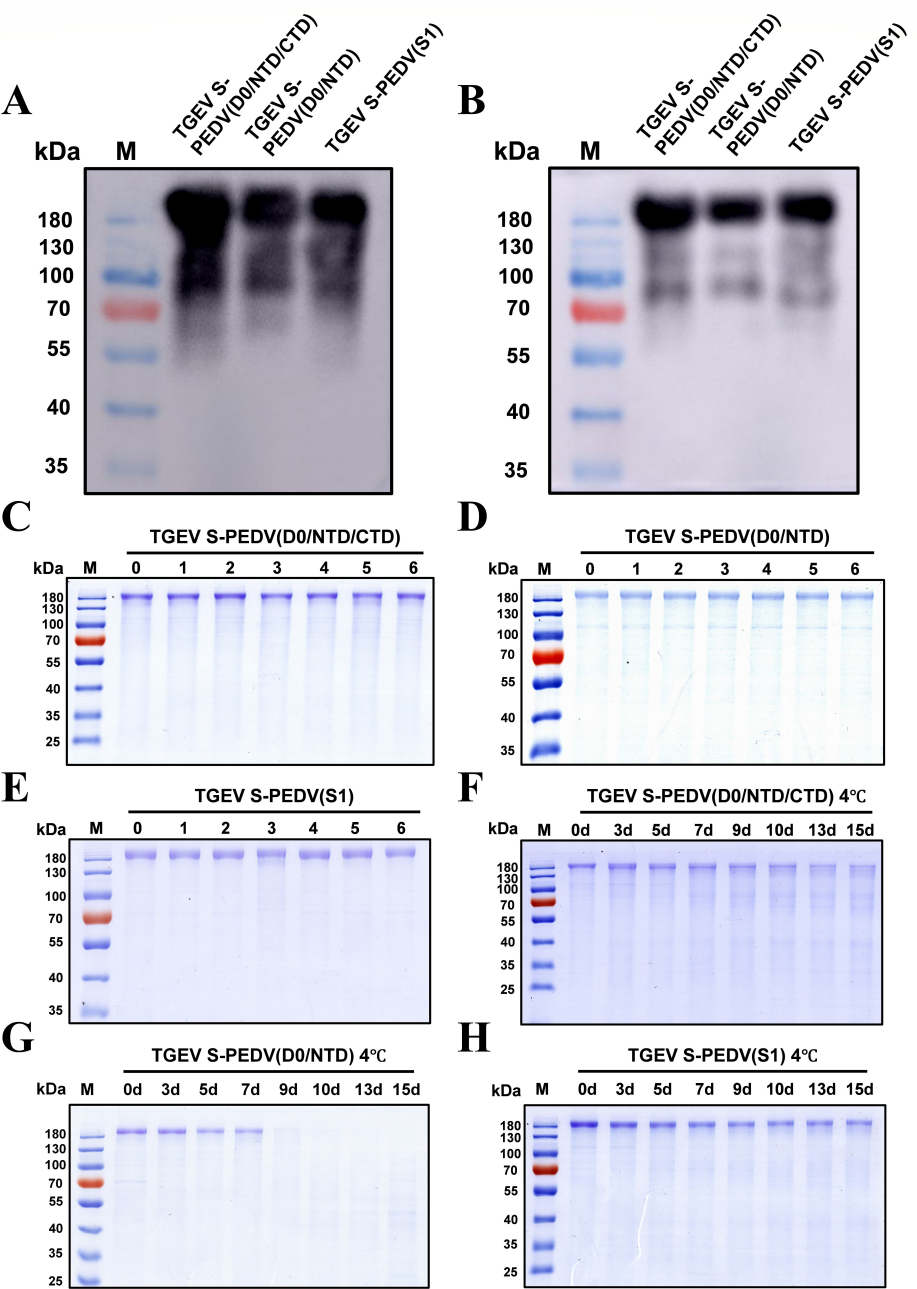
Immunogenicity and stability analysis of bivalent chimeric S protein candidates. (**A and B**) Identification of the immunogenicity of chimeric S proteins by western blotting. The different chimeric S proteins were detected using TGEV S-positive serum (**A**) and PEDV S-positive serum (**B**) as primary antibodies. (**C–E**) Determination of the freeze‒thaw stability of chimeric S proteins by SDS-PAGE. All the proteins were subjected to six freeze‒thaw cycles. (**F–H**) Stability determination of chimeric S proteins stored at 4°C by SDS-PAGE. All the proteins were stored at 4°C for 15 days, and the samples were analyzed on days 0, 3, 5, 7, 9, 10, 13, and 15.

To explore the immunogenicity of these three chimeras, a western blot analysis was conducted with serum from mice immunized with TGEV S or PEDV S as the primary antibody. As expected, all three spike protein chimeras demonstrated specific reactivity with both TGEV S- and PEDV S-positive sera (Fig. 6A and B). In addition, the stability of these three spike protein chimeras was assessed under the following conditions: (i) short-term stability (15 days of storage at 4°C) and (ii) freeze‒thaw stability (six freeze‒thaw cycles at −80°C). As depicted in Fig. 6C through E, SDS-PAGE analysis revealed that there was no significant degradation after six freeze‒thaw cycles in all three spike protein chimeras. Moreover, all three chimeras could be stored at 4°C for at least 7 days without observable degradation (Fig. 6F through H). In detail, TGEV S-PEDV(D0/NTD) can be stored at 4°C for 7 days, and TGEV S-PEDV(D0/NTD/CTD) and TGEV S-PEDV(S1) can be stored at 4°C for at least 15 days. Together, these results indicate that TGEV S-PEDV(D0/NTD), TGEV S-PEDV(D0/NTD/CTD), and TGEV S-PEDV(S1) exhibit promising immunogenicity, short-term stability, and freeze‒thaw stability.

### The chimeric protein [TGEV S-PEDV(D0/NTD)] can induce neutralizing antibodies against both TGEV and PEDV in mice

**Fig 7:**
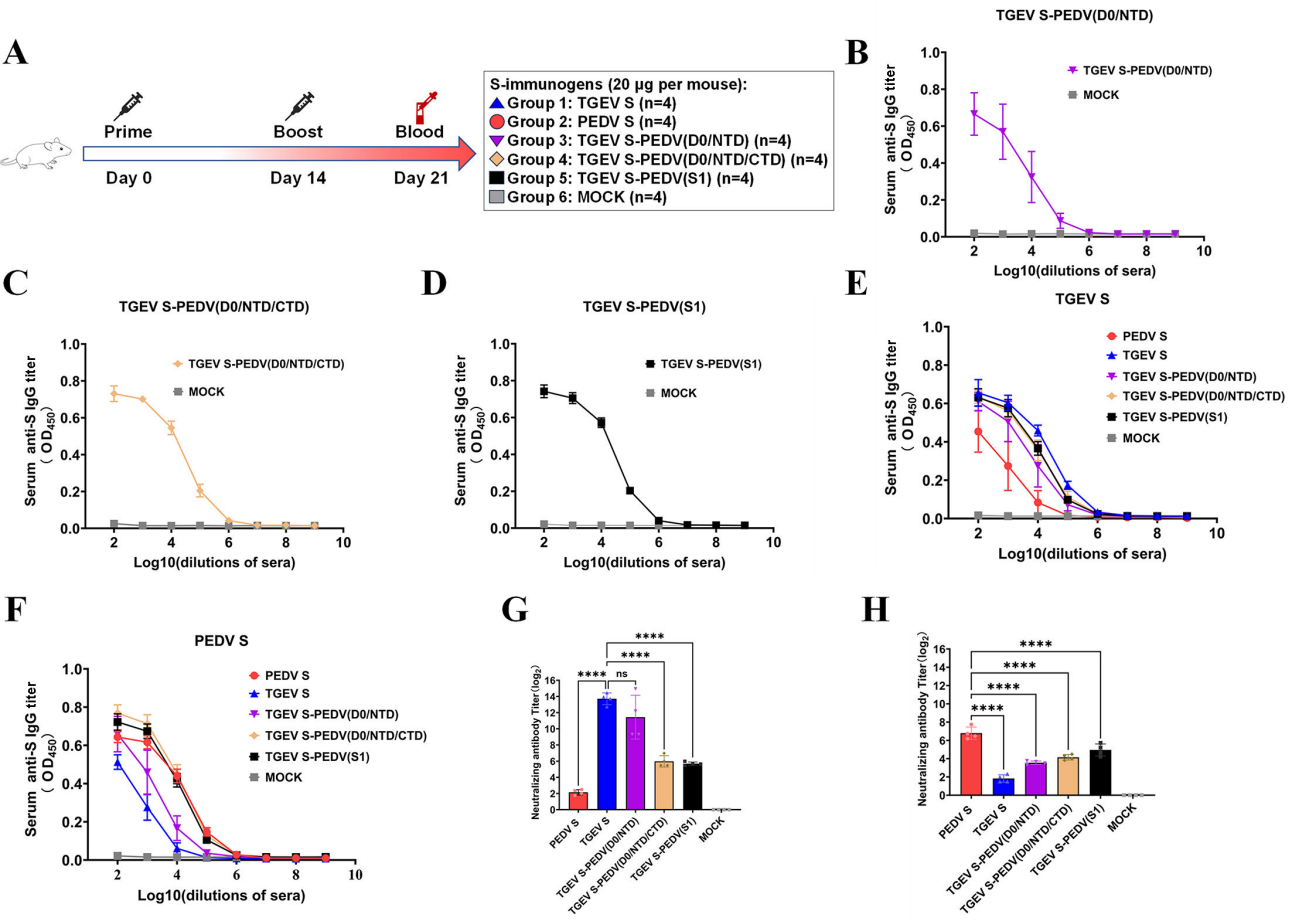
Evaluation of the immunogenicity of chimeric S proteins in mice. (**A**) Experimental design for mouse vaccination. (**B–D**) Serum IgG against different chimeric S proteins, including TGEV S-PEDV(D0/NTD) (**B**), TGEV S-PEDV(D0/NTD/CTD) (**C**), and TGEV S-PEDV(S1) (**D**), was detected by ELISA. (**E and F**) The levels of TGEV S (**E**) and PEDV S (**F**) protein-binding IgG antibodies in the serum were measured at 21 d by ELISA. (**G and H**) Serum-neutralizing antibodies against TGEV (**G**) and PEDV (**H**) were detected. The data are presented as the means ± SDs of four mice per group. The asterisks in the figures indicate significant differences (****P < 0.0001; ns, not significant).

We next assessed the immunogenicity of chimeric S proteins in a mouse model. The study design and experimental groups are summarized in Fig. 7A: group 1 was inoculated with TGEV S (*n* = 4), group 2 was inoculated with PEDV S (*n* = 4), group 3 was inoculated with TGEV S-PEDV(D0/NTD) (*n* = 4), group 4 was inoculated with TGEV S-PEDV(D0/NTD/CTD) (*n* = 4), group 5 was inoculated with TGEV S-PEDV(S1) (*n* = 4), and group 6 was inoculated with normal saline as a mock. The mice were inoculated by the same protocol as described above. Serum samples were collected to evaluate the antibody responses using enzyme-linked immunosorbent assay (ELISA) or virus neutralization tests. As shown in Fig. 7B through D, all three chimeric S proteins were capable of eliciting a robust IgG immune response. Similar to the results above, all three chimeric S proteins demonstrated excellent immunogenicity. Furthermore, we determined the TGEV S- and PEDV S-specific IgG titers in sera from three chimeric S protein-immunized mice. As shown in Fig. 7E and F, all three chimeric S proteins induced TGEV S- and PEDV S-specific IgG antibodies in the serum.

We subsequently measured the neutralizing antibodies against TGEV and PEDV in the sera of the three chimeric S protein-immunized mice. As shown in Fig. 7G, the mean neutralizing antibody titers against TGEV in the TGEV S-PEDV(D0/NTD) group were similar to those in the TGEV S group, whereas the mean neutralizing antibody titers against TGEV in the TGEV S-PEDV(D0/NTD/CTD) group and in the TGEV S-PEDV(S1) group were lower than those in the TGEV S group, but these titers were still higher than those in the PEDV S group, indicating a partial neutralizing protective capacity. As shown in Fig. 7H, although the level of neutralization against PEDV in the three chimeric S protein groups was lower than that in the PEDV S group, the mean neutralizing antibody titers against PEDV induced by all the three chimeric S protein groups were greater than those in the TGEV S group. These results suggest that the three chimeric S proteins could effectively induce varying levels of neutralizing antibodies against both TGEV and PEDV in mice.

### The chimeric protein [TGEV S-PEDV(D0/NTD)] can induce neutralizing antibodies against both TGEV and PEDV in piglets

**Fig 8:**
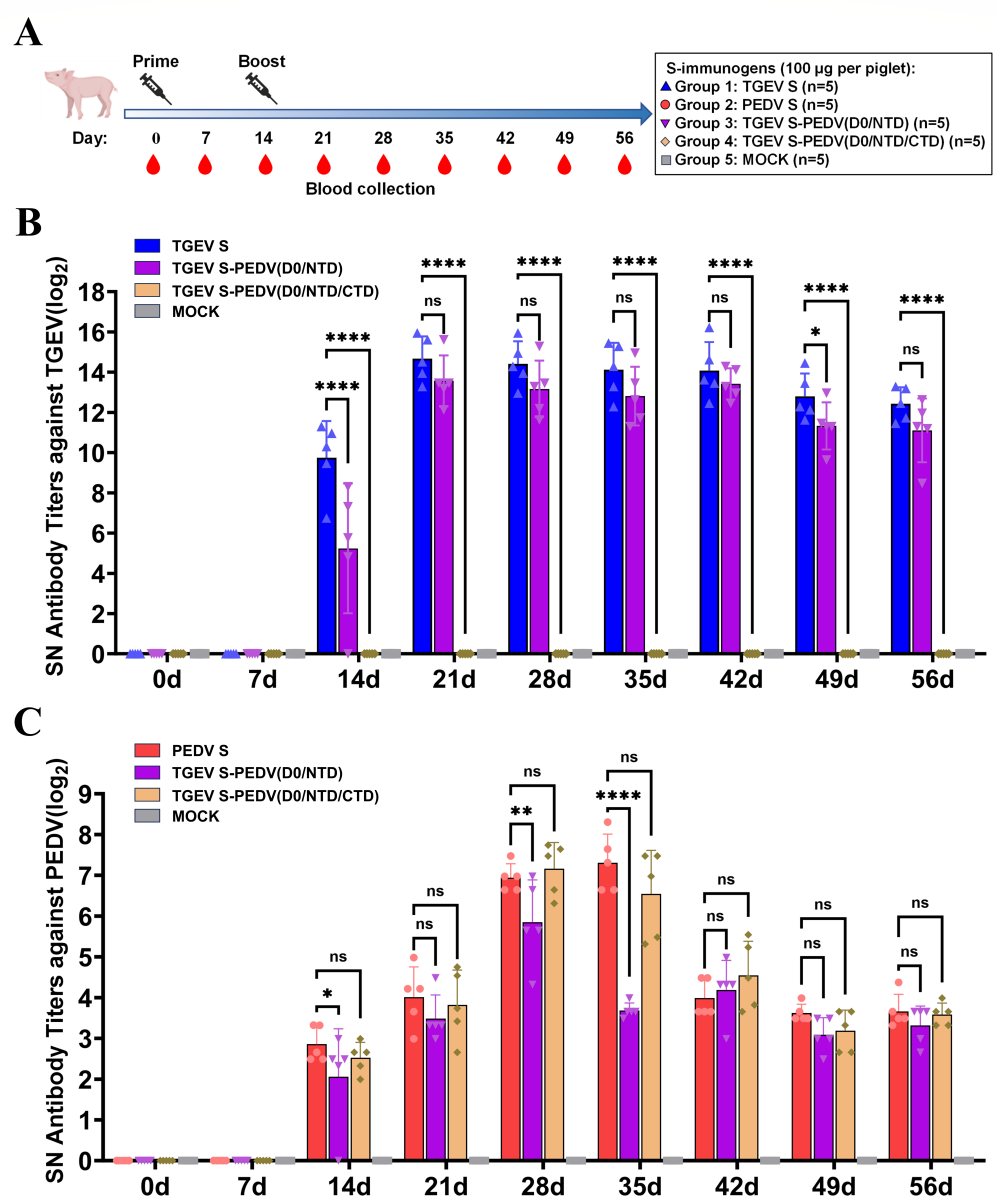
Evaluation of the immunogenicity of chimeric S proteins in piglets. (**A**) Experimental design for piglet vaccination, including immune procedures and specimen collection schedule. (**B and C**) Detection of specific neutralizing antibody levels against TGEV (**B**) and PEDV (**C**) in the sera of piglets immunized with chimeric S proteins on different days postvaccination. The data are presented as the means ± SDs of five piglets per group. The significant differences are indicated as follows: ns: not significant; *P < 0.05, **P < 0.01, ****P < 0.0001.

Based on the results obtained from mouse experiments, we selected TGEV S-PEDV(D0/NTD) and PEDV(D0/NTD/CTD) for subsequent immunogenicity evaluation in piglets. The study design is summarized in Fig. 8A. Briefly, five groups of 2-month-old piglets born from TGEV- and PEDV-negative sows were vaccinated intramuscularly with chimeric S proteins [TGEV S, PEDV S, TGEV S-PEDV(D0/NTD), TGEV S-PEDV(D0/NTD/CTD)] or normal saline as a mock and then administered a booster immunization 2 weeks later. Serum samples were collected at weekly intervals until 8 weeks (0, 7, 14, 21, 28, 35, 42, 49, and 56 days postvaccination, dpv) after which the neutralizing antibody titers against TGEV and PEDV were determined. Analysis of the neutralizing antibody titers revealed that piglets immunized with TGEV S exhibited a significantly high level of neutralizing antibodies against TGEV after booster immunization (14 dpv) and increased rapidly to the average peak titer of approximately 2^14.7^ at 21 dpv (Fig. 8B), whereas the neutralizing antibodies against PEDV reached a peak titer of approximately 2^7.3^ at 35 dpv in the PEDV S group (Fig. 8C). Furthermore, we determined the neutralizing antibody titers against TGEV and PEDV in the sera of these chimeric S protein-immunized piglets. As shown in Fig. 8B and C, the TGEV neutralizing antibody levels in the TGEV S-PEDV(D0/NTD) group were close to those in the TGEV S group, with an average peak titer of approximately 2^13.6^ at 21 dpv. Moreover, the TGEV S-PEDV(D0/NTD) group presented sufficient neutralizing antibodies against PEDV, with an average peak titer of approximately 2^5.8^ at 28 dpv. In addition, the PEDV neutralizing antibody levels in the TGEV S-PEDV(D0/NTD/CTD) group were close to those in the PEDV S group, with an average peak titer of approximately 2^7.2^ at 28 dpv, whereas the TGEV S-PEDV(D0/NTD/CTD) group presented no neutralizing antibodies against TGEV (Fig. 8B and C). Together, these results showed that TGEV S-PEDV(D0/NTD) induced broad-spectrum neutralizing antibodies against TGEV and PEDV.

### Design strategy for a multivalent single antigen targeting the S proteins of PEDV and TGEV

In the swine industry, diarrhea caused by TGEV and PEDV causes serious economic losses worldwide. Thus, the development of protective vaccines against TGEV and PEDV remains a top priority. Recently, subunit vaccines based on the trimeric ectodomain of the S protein were developed and demonstrated favorable immune effects in mice and piglets (35, 39, 40). In this study, we first obtained trimeric full-length TGEV S and PEDV S and demonstrated that the immunogenic efficacy of TGEV S is superior to that of PEDV S, indicating that the TGEV S protein in its trimeric form can be used as a backbone to design a TGEV/PEDV S chimeric protein (Fig. 2). Furthermore, we predicted the potential B-cell epitopes for TGEV S and PEDV S, and the results revealed that the distribution of potential B-cell epitopes in TGEV S was similar to that in PEDV S. The epitopes of TGEV S and PEDV S were located mainly in S1-D0, S1-NTD, and S1-CTD (Fig. 3 and 4). Therefore, the corresponding segments were replaced on TGEV S with different domains from those of PEDV S1; thus, seven distinct chimeric S proteins aimed at the formation of a multiepitope single antigen were designed. By comparing their expression levels, we subsequently screened three chimeric S proteins, TGEV S-PEDV(D0/NTD), TGEV S-PEDV(D0/NTD/CTD), and TGEV S-PEDV(S1). We speculate that the composition of these three chimeric S proteins may have a lesser impact on the stability of the S trimer protein structure and that these three chimeric S proteins exhibited high short-term stability and freeze‒thaw stability (Fig. 6).

**Fig 9:**
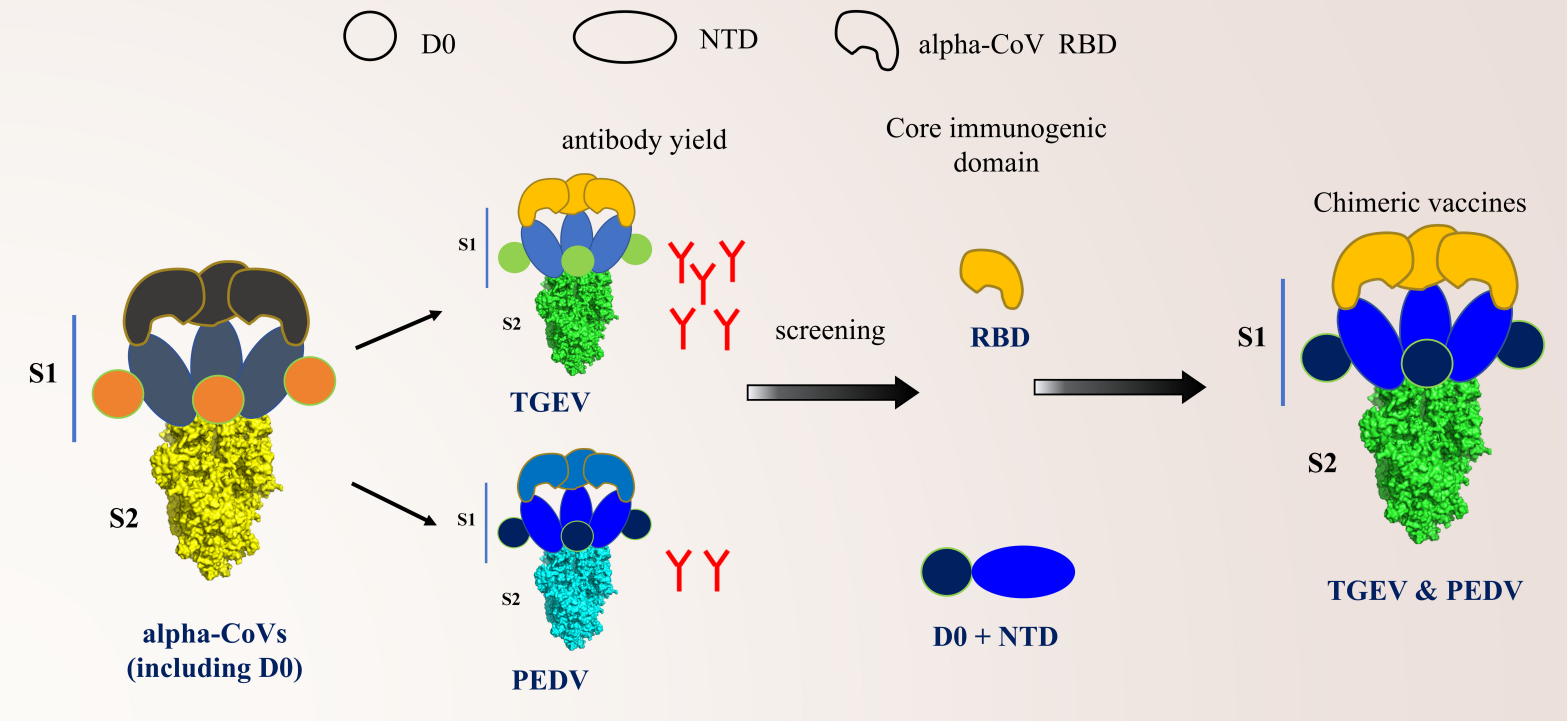
Research and development model of bivalent chimeric S protein for TGEV and PEDV. The model revealed that among alpha-coronaviruses harboring the D0 domain, including TGEV and PEDV, TGEV induces significantly higher levels of neutralizing antibodies than does PEDV. Through systematic screening, we identified the RBD as the core immunogenic domain of TGEV, whereas the D0 + NTD domain constitutes the core immunogenic domain of PEDV. Thus, we engineered a chimeric S protein using TGEV S as the backbone framework. The S1 subunit of the chimeric S protein comprises the PEDV D0 + NTD domain and the TGEV RBD domain. This chimeric S protein can induce broad-spectrum neutralizing antibodies against both TGEV and PEDV.

Neutralizing antibodies are critical indicators for evaluating their immune protective effect since they can directly reflect the protective capacity of a vaccine (41, 42), and broad-spectrum activity is also a key factor in the design of an effective vaccine. In a mouse model, three chimeric S proteins elicited differential neutralizing antibody responses against both TGEV and PEDV. Notably, TGEV S-PEDV(D0/NTD) induced neutralizing antibody levels against TGEV comparable to those of the full-length TGEV S, whereas TGEV S-PEDV(D0/NTD/CTD) and TGEV S-PEDV(S1) showed significantly lower induction (Fig. 7). We further determined the neutralizing activity of the antibodies in the serum of immunized piglets. The results showed that TGEV S-PEDV(D0/NTD) could produce broad-spectrum neutralizing antibodies against both TGEV and PEDV. Considering that TGEV S-PEDV(D0/NTD/CTD) did not produce neutralizing antibodies against TGEV, we surmised that TGEV RBD played a crucial role in inducing neutralizing antibodies, which was confirmed in a mouse model (Fig. 8). Accordingly, we speculate that replacing the corresponding segments on TGEV S with D0-NTD domains from PEDV S is an effective strategy for designing novel bivalent subunit vaccines (Fig. 9).

In summary, on the basis of our research results, we propose a research and development strategy for producing efficient broad-spectrum subunit vaccines against both TGEV and PEDV. Replacing the corresponding segments on TGEV S with D0-NTD domains from PEDV S does not affect the protein expression level. Furthermore, through immunogenicity evaluation in mice and weaned piglets, it was confirmed that TGEV S-PEDV(D0/NTD) can provide broad-spectrum cross-neutralization protection against TGEV and PEDV. Our findings provide a novel strategy for the development of safe and effective bivalent subunit vaccine candidates against TGEV and PEDV in the future.

## MATERIALS AND METHODS

### Cell lines and viruses

Vero cells (CCL-81) and PK-15 cells were obtained from the American Type Culture Collection (ATCC) and were cultured in Dulbecco’s modified Eagle medium (DMEM; Gibco, USA) supplemented with 10% fetal bovine serum (FBS), 100 U/mL penicillin, and 100 µg/mL streptomycin at 37°C in a 5% CO_2_ humidified atmosphere. HEK293F cells preserved in our laboratory were maintained in serum-free Union 293 medium (Union, Cat# UP1000) with shaking at 120 rpm and 37°C in a humidified atmosphere comprising 8% CO_2_. The PEDV G2 strain CT P10 (GenBank accession no. MN114121) was propagated in Vero cells supplemented with 5 µg/mL trypsin (Gibco). The TGEV strain WH-1 (GenBank accession no. HQ462571) was propagated in PK-15 cells in Dulbecco’s modified Eagle’s medium (DMEM; Gibco, USA) supplemented with 10% fetal bovine serum (FBS) in an incubator.

### B-cell epitope prediction analysis

Homology modeling of the S protein of PEDV CT P10 and TGEV WH-1 was conducted using the SWISS-MODEL (http://swissmodel.expasy.org/) to perform B-cell epitope predictions of the S-trimers (1). The cryo-electron microscopy (cryo-EM) structure of the PEDV PT52 S-trimer (PDB ID: 7W6M) was used as a template.

According to previous research, B-cell epitopes were predicted and analyzed (IEDB, https://www.iedb.org/). Briefly, structure-based B-cell epitopes were predicted using DiscoTope 3.0 (https://services.healthtech.dtu.dk/services/DiscoTope-3.0/) with a confidence threshold of 0.90 (recall up to ~50%, default). For linear B-cell epitope prediction, ABCpred, the IEDB server, and BcePred were employed with default parameters to optimize the sensitivity, specificity, and positive predictive value (43–45). To improve the accuracy of epitope prediction, the results of these three prediction sites were synthesized, and the overlapping part of the epitopes predicted by at least two websites was selected as the linear epitope of the dominant B cells. All the predicted residues were labeled in the corresponding structures using PyMOL (Schrödinger LLC). No epitope was predicted within the signal peptide (SP), FP, or TM domains of the two S proteins.

### Plasmid construction, protein expression, and purification

The human-optimized codon gene encoding the PEDV spike protein (GenBank accession no. QHB92364.1) and the human-optimized codon gene encoding the TGEV spike protein (GenBank accession no. ADY39740.1) were synthesized (GenScript Biotech). The DNA sequences of PEDV S (residues 1–1,319) and TGEV S ectodomain (residues 1–1,388) were cloned and inserted into a pCAGGS vector with a C-terminal GCN4 trimerization motif followed by a Strep-tag and an 8 × HisTag sequence. The other eukaryotic expression plasmids were constructed as shown in Fig. 5A. All S proteins were stabilized with a dual-proline (2P) mutation (^1076^SL^1077^ → ^1076^PP^1077^ for PEDV S, ^1139^EL^1140^ → ^1139^PP^1140^ for TGEV S). The expression plasmid was transiently transfected into a suspension of HEK293F cells using polyetherimide (PEI), and the cells were cultured for another 6 days at 37°C and 8% CO_2_. The proteins were harvested from the supernatants of the cell culture medium and purified on a Ni-NTA column. After affinity purification, the proteins were concentrated and subsequently subjected to additional purification using a size exclusion chromatography (SEC) column (Superose 6 increase 10/300 Gl; GE Healthcare, U.S.A.), utilizing a running buffer consisting of 20 mM HEPES (pH 7.6) and 150 mM NaCl. All the proteins were exchanged in phosphate-buffered saline (PBS) and stored at −80°C.

### SDS–PAGE and western blotting

SDS-PAGE and western blotting were carried out as described by us earlier (46). The collected protein samples were mixed with 5 × reducing loading buffer, boiled for 10 min, and then loaded onto 10% SDS‒PAGE gels. The proteins were electrophoresed for 2.5 h at 80 V in a Bio-Rad MINI-PROTEAN Tetra system (Bio-Rad Laboratories). The gel was stained with Coomassie Brilliant Blue R-250 (Bio-Rad) for 30 min at room temperature and then decolorized with eluent overnight. For western blotting, after the proteins were resolved by SDS‒PAGE, they were transferred onto a polyvinylidene fluoride (PVDF) membrane. The PVDF membranes were blocked with 5% skim milk/TBST overnight at 4°C. The membrane was then incubated with diluted mouse serum (1:500 dilution) or an anti-His-tag monoclonal antibody (1:5,000 dilution) for 2 h at room temperature. The membranes were subsequently incubated with a goat anti-mouse secondary antibody (Abbkine, no. A21010) for 1 h (1:5.000) at room temperature. Finally, the membranes were visualized using an enhanced chemiluminescence system (Amersham Imager 600, GE Healthcare).

### Electron microscopy

For negative staining experiments, the spike protein samples were directly analyzed using a Talos L120C G2 transmission electron microscope (Thermo Fisher Scientific). Copper grids with 300 mesh and a thin layer of continuous carbon floated on top (EM Sciences) were glow-discharged at 15 mA for 60 s. The spike protein samples were diluted to 0.005 mg/mL, and 5 µL was applied to the grids for 30 s. Then, three drops containing 10  µL of uranyl acetate (3%, vol/vol) staining solution were applied to the parafilm (Bemis). Samples on the grids were then stained with the third drop of uranyl acetate for 60 s, followed by allowing it to rapidly combine with the first two drops. The specimen was finally gently blotted from the side with filter paper, air-dried at 25 °C for 30 min, and stored until imaging.

For cryo-EM sample preparation, 3 µL of spike protein solution (1 mg/mL) was applied to a glow-discharged (40 s, 15 mA) holey carbon Cu Quantifoil grid (R1.2/1.3, 300 mesh). The grids were blotted for 3 s at 100% humidity and 280 K and then plunge-frozen in liquid ethane using a Vitrobot (Thermo-Scientific). Cryo-EM micrographs with a defocus from −1 to −2  µm were collected on a Gatan K3 direct electron detector in superresolution mode on a Krios G4 cryoTEM (Thermo Fisher). A series of micrographs was acquired at ×105,000 magnification with a pixel size of 1.648  Å pixel^−1^ and a dose of 50 e^−^ Å^−2^ for sample quality assessment. Automated data collection was performed by EPU software (Thermo Fisher Scientific).

### Design of the mouse vaccination experiments

Female BALB/c mice (four per group) aged 6 weeks were immunized with different proteins at 0 and 2 weeks. Proteins (20 µg) diluted in normal saline were mixed 1:1 with QuickAntibody-Mouse3W adjuvant (BioDragon). The mice were intramuscularly inoculated with 100 µL of this solution (50 µL into each hind leg). One week after the final immunization, sera were collected for subsequent assays.

### Design of the pig vaccination experiment

To determine the efficacy of the chimeric S proteins, 25 2-month-old PEDV/TGEV-naive piglets were placed in separate rooms and randomly assigned to four experimental groups and one mock group: the PEDV S-immunized group (*n* = 5), the TGEV S-immunized group (*n* = 5), the TGEV S-PEDV(D0/NTD)-immunized group (*n* = 5), the TGEV S-PEDV(D0/NTD/CTD)-immunized group (*n* = 5), and the PBS-immunized group (*n* = 5). The piglets in the immunization groups were injected intramuscularly with the S vaccines (100 µg per piglet) or with PBS. At 14 dpv, the piglets in the immunization groups received a booster dose of the vaccine. All vaccines were formulated in Montanide ISA 201 VG adjuvant. Blood samples were collected from the anterior vena cava weekly. Each serum sample was tested for SN titers against PEDV and TGEV.

### Enzyme-linked immunosorbent assay (ELISA)

Serum S-specific antibodies in each group were detected at 21 DPI. Titers of S-specific antibodies in the serum were determined via indirect ELISA with purified S protein as the antigen, as described previously (38). Briefly, ELISA plates were coated with purified PEDV or TGEV S protein at 0.1  µM/well in citrate-buffered saline (CBS, pH 9.6) overnight at 4°C and subsequently blocked with 1% (wt/vol) bovine serum albumin (BSA) in phosphate-buffered saline (PBS) containing 0.05% Tween 20 (PBST) at 37°C. For antibody detection, the plates were incubated with 10-fold serially diluted sera for 1 h at 37°C. After standard washes, 100 µL of diluted horseradish peroxidase (HRP)-conjugated goat anti-mouse IgG (Boster, Wuhan, China) was used as the secondary antibody, and 3,3′,5,5′-tetramethylbenzidine (TMB) (Beyotime) was used as the substrate for detection. The final absorbance [optical density (OD)] was measured at 450 nm and 630  nm using a Spark 10 M microplate reader (Tecan) after the reaction was stopped with 2 M H_2_SO_4_. Serum from mice immunized with normal saline was used as a control.

### Neutralization assays

To detect PEDV-specific neutralizing antibodies, the serum-neutralizing antibody titers were determined with a virus neutralization test in 96-well cell culture plates (46). Briefly, the serum samples were heat-inactivated at 56°C for 30 min and serially diluted twofold with DMEM. The diluted samples were mixed with an equal volume of 200 TCID_50_ of PEDV and incubated at 37°C for 1 h. Subsequently, 100 µL of the virus–serum mixture was inoculated into Vero cells in 96-well plates at 37°C for 1 h, followed by the addition of 100 µL of DMEM with 5 µg/mL trypsin, and the mixture was maintained at 37°C in a 5% CO_2_ incubator. After incubation for 24–48 h, the neutralizing antibody titers were expressed as the reciprocal of the highest serum dilution that inhibited PEDV-specific CPE.

To detect TGEV-specific neutralizing antibodies, the serum-neutralizing antibody titers were determined with a virus neutralization test in 96-well cell culture plates. Briefly, the serum samples were heat-inactivated at 56°C for 30 min and serially diluted 2-fold with DMEM. The diluted samples were mixed with an equal volume of TGEV (200 TCID_50_) and incubated at 37°C for 1 h. Subsequently, 100 µL of the virus-serum mixture was inoculated into PK-15 cells in 96-well plates. After incubation at 37°C for 48–72 h, the neutralizing antibody titers were expressed as the reciprocal of the highest serum dilution that inhibited TGEV-specific CPE.

### Statistical analysis

Statistical analysis was carried out using GraphPad Prism 8.0. Statistical significance was determined using an unpaired two-tailed Student’s *t* test. The Data are presented as the means ± SDs (95% confidence intervals). *, *P*  <  0.05 was considered statistically significant, **, *P*  <  0.01 was considered highly significant, and ****, *P*  <  0.0001 was considered extremely significant. All experiments were further confirmed using biological replicates.

## Data Availability

The data that support the findings of this study are openly available in this article and are available from the corresponding author after request.

## References

[1] Boniotti MB, Papetti A, Lavazza A, Alborali G, Sozzi E, Chiapponi C, Faccini S, Bonilauri P, Cordioli P, Marthaler D. 2016. Porcine epidemic diarrhea virus and discovery of a recombinant swine enteric coronavirus, Italy. Emerg Infect Dis 22:83–87. doi:10.3201/eid2201.150544 26689738 PMC4696687

[2] Gu JP, Yue XW, Xing R, Li CY, Yang ZB. 2012. Progress in genetically engineered vaccines for porcine transmissible gastroenteritis virus. Rev Med Vet (Toulouse) 163:107–111.

[3] Hanke D, Jenckel M, Petrov A, Ritzmann M, Stadler J, Akimkin V, Blome S, Pohlmann A, Schirrmeier H, Beer M, Höper D. 2015. Comparison of porcine epidemic diarrhea viruses from Germany and the United States, 2014. Emerg Infect Dis 21:493–496. doi:10.3201/eid2103.141165 25695311 PMC4344272

[4] Lee C. 2015. Porcine epidemic diarrhea virus: an emerging and re-emerging epizootic swine virus. Virol J 12:193. doi:10.1186/s12985-015-0421-2 26689811 PMC4687282

[5] Pearce SC, Schweer WP, Schwartz KJ, Yoon KJ, Lonergan SM, Gabler NK. 2016. Pig jejunum protein profile changes in response to a porcine epidemic diarrhea virus challenge. J Anim Sci 94:412–415. doi:10.2527/jas.2015-981526812347

[6] Sun RQ, Cai RJ, Chen YQ, Liang PS, Chen DK, Song CX. 2012. Outbreak of porcine epidemic diarrhea in suckling piglets, China. Emerg Infect Dis 18:161–163. doi:10.3201/eid1801.111259 22261231 PMC3381683

[7] Ahmad ZA, Yeap SK, Ali AM, Ho WY, Alitheen NBM, Hamid M. 2012. scFv antibody: principles and clinical application. Clin Dev Immunol 2012:980250. doi:10.1155/2012/980250 22474489 PMC3312285

[8] Xia L, Yang Y, Wang J, Jing Y, Yang Q. 2018. Impact of TGEV infection on the pig small intestine. Virol J 15:102. doi:10.1186/s12985-018-1012-9 29914507 PMC6006930

[9] Li W, Li H, Liu Y, Pan Y, Deng F, Song Y, Tang X, He Q. 2012. New variants of porcine epidemic diarrhea virus, China, 2011. Emerg Infect Dis 18:1350–1353. doi:10.3201/eid1808.120002 22840964 PMC3414035

[10] Zhang H, Zou C, Peng O, Ashraf U, Xu Q, Gong L, Fan B, Zhang Y, Xu Z, Xue C, Wei X, Zhou Q, Tian X, Shen H, Li B, Zhang X, Cao Y. 2023. Global dynamics of porcine enteric coronavirus PEDV epidemiology, evolution, and transmission. Mol Biol Evol 40:msad052. doi:10.1093/molbev/msad052 36869744 PMC10027654

[11] Lee S, Lee C. 2014. Outbreak-related porcine epidemic diarrhea virus strains similar to US strains, South Korea, 2013. Emerg Infect Dis 20:1223–1226. doi:10.3201/eid2007.140294 24960370 PMC4073847

[12] Stevenson GW, Hoang H, Schwartz KJ, Burrough ER, Sun D, Madson D, Cooper VL, Pillatzki A, Gauger P, Schmitt BJ, Koster LG, Killian ML, Yoon KJ. 2013. Emergence of Porcine epidemic diarrhea virus in the United States: clinical signs, lesions, and viral genomic sequences. J Vet Diagn Invest 25:649–654. doi:10.1177/1040638713501675 23963154

[13] Lee KM, Moro M, Baker JA. 1954. Transmissible gastroenteritis in pigs. J Am Vet Med Assoc 124:294. 13142978

[14] Paton D, Ibata G, Sands J, McGoldrick A. 1997. Detection of transmissible gastroenteritis virus by RT-PCR and differentiation from porcine respiratory coronavirus. J Virol Methods 66:303–309. doi:10.1016/s0166-0934(97)00055-4 9255741 PMC7119849

[15] Cubero MJ, León L, Contreras A, Astorga R, Lanza I, Garcia A. 1993. Transmissible gastroenteritis in pigs in south east Spain: prevalence and factors associated with infection. Vet Rec 132:238–241. doi:10.1136/vr.132.10.238 8384735

[16] Hess RG, Bollwahn W, Pospischil A, Heinritzi K, Bachmann PA. 1980. Current aspects in the etiology of viral diarrheas of swine: occurrence of infections with the epizootic viral diarrhea (EVD) virus. Berl Munch Tierarztl Wochenschr 93:445–449. 6258554

[17] Lin CM, Gao X, Oka T, Vlasova AN, Esseili MA, Wang QH, Saif LJ. 2015. Antigenic relationships among porcine epidemic diarrhea virus and transmissible gastroenteritis virus strains. J Virol 89:3332–3342. doi:10.1128/JVI.03196-14 25589635 PMC4337547

[18] Laude H, Rasschaert D, Delmas B, Godet M, Gelfi J, Charley B. 1990. Molecular biology of transmissible gastroenteritis virus. Vet Microbiol 23:147–154. doi:10.1016/0378-1135(90)90144-k 2169670 PMC7117338

[19] Brian DA, Baric RS. 2005. Coronavirus genome structure and replication. Curr Top Microbiol Immunol 287:1–30. doi:10.1007/3-540-26765-4_1 15609507 PMC7120446

[20] Kong F, Jia H, Xiao Q, Fang L, Wang Q. 2023. Prevention and control of swine enteric coronaviruses in China: a review of vaccine development and application. Vaccines (Basel) 12:11. doi:10.3390/vaccines12010011 38276670 PMC10820180

[21] Chen F, Knutson TP, Rossow S, Saif LJ, Marthaler DG. 2019. Decline of transmissible gastroenteritis virus and its complex evolutionary relationship with porcine respiratory coronavirus in the United States. Sci Rep 9:3953. doi:10.1038/s41598-019-40564-z 30850666 PMC6408454

[22] Huang C-Y, Draczkowski P, Wang Y-S, Chang C-Y, Chien Y-C, Cheng Y-H, Wu Y-M, Wang C-H, Chang Y-C, Chang Y-C, Yang T-J, Tsai Y-X, Khoo K-H, Chang H-W, Hsu S-TD. 2022. In situ structure and dynamics of an alphacoronavirus spike protein by cryo-ET and cryo-EM. Nat Commun 13:4877. doi:10.1038/s41467-022-32588-3 35986008 PMC9388967

[23] Wrapp D, McLellan JS. 2019. The 3.1-Angstrom cryo-electron microscopy structure of the porcine epidemic diarrhea virus spike protein in the prefusion conformation. J Virol 93:e00923-19. doi:10.1128/JVI.00923-19 31534041 PMC6854500

[24] Kirchdoerfer RN, Bhandari M, Martini O, Sewall LM, Bangaru S, Yoon KJ, Ward AB. 2021. Structure and immune recognition of the porcine epidemic diarrhea virus spike protein. Structure 29:385–392. doi:10.1016/j.str.2020.12.003 33378641 PMC7962898

[25] Li F. 2016. Structure, function, and evolution of coronavirus spike proteins. Annu Rev Virol 3:237–261. doi:10.1146/annurev-virology-110615-042301 27578435 PMC5457962

[26] Li W, van Kuppeveld FJM, He Q, Rottier PJM, Bosch B-J. 2016. Cellular entry of the porcine epidemic diarrhea virus. Virus Res 226:117–127. doi:10.1016/j.virusres.2016.05.031 27317167 PMC7114534

[27] Li C, Li W, Lucio de Esesarte E, Guo H, van den Elzen P, Aarts E, van den Born E, Rottier PJM, Bosch B-J. 2017. Cell attachment domains of the porcine epidemic diarrhea virus spike protein are key targets of neutralizing antibodies. J Virol 91:e00273-17. doi:10.1128/JVI.00273-17 28381581 PMC5446644

[28] Ma Z, Li Z, Li Y, Zhao X, Zheng C, Li Y, Guo X, Xu L, Zheng Z, Liu G, Zheng H, Xiao S. 2025. Changes in the motifs in the D0 and SD2 domains of the S protein drive the evolution of virulence in enteric coronavirus porcine epidemic diarrhea virus. J Virol 99:e0209224. doi:10.1128/jvi.02092-24 40035514 PMC11998522

[29] Zhang L, Liu JB, Liu HZ, Lian YX, Huang YW, Cong F. 2024. The emergence of novel variants of the porcine epidemic diarrhea virus spike gene from 2011 to 2023. Transbound Emerg Dis 2024:2876278. doi:10.1155/2024/2876278 40303057 PMC12017180

[30] Li Y, Yang S, Qian J, Liu S, Li Y, Song X, Cao Q, Guo R, Zhao Y, Sun M, Hu M, Li J, Zhang X, Fan B, Li B. 2025. Molecular characteristics of the immune escape of coronavirus PEDV under the pressure of vaccine immunity. J Virol 99:e0219324. doi:10.1128/jvi.02193-24 40237499 PMC12090811

[31] Du PF, Yan QH, Zhang XA, Zeng WJ, Xie KY, Yuan ZM, Liu XD, Liu XY, Zhang LH, Wu KK, Li XW, Fan SQ, Zhao MQ, Chen JD. 2023. Virus-like particle vaccines with epitopes from porcine epidemic virus and transmissible gastroenteritis virus incorporated into self-assembling ADDomer platform provide clinical immune responses in piglets. Front Immunol 14. doi:10.3389/fimmu.2023.1251001 PMC10628522 37942329

[32] Fan L, Yi X, Zhong C, Yang C, Niu Z, Xue X, Wang W, Guo R, Ma J, Zha Y, Shu J, Li J, Li B. 2025. A trivalent enteric coronaviruses inactivated vaccine provides effective protection against PEDV, TGEV, and PDCoV. Vet Microbiol 308:110630. doi:10.1016/j.vetmic.2025.110630 40633274

[33] Song D, Moon H, Kang B. 2015. Porcine epidemic diarrhea: a review of current epidemiology and available vaccines. Clin Exp Vaccine Res 4:166–176. doi:10.7774/cevr.2015.4.2.166 26273575 PMC4524901

[34] Li X, Li Y, Huang J, Yao Y, Zhao W, Zhang Y, Qing J, Ren J, Yan Z, Wang Z, Hu X, Kang D, Liu H, Yan Z. 2022. Isolation and oral immunogenicity assessment of porcine epidemic diarrhea virus NH-TA2020 strain: one of the predominant strains circulating in China from 2017 to 2021. Virol Sin 37:646–655. doi:10.1016/j.virs.2022.08.002 35961502 PMC9583181

[35] Xu M, Yang Z, Yang N, Li H, Ma H, Yi J, Hou H, Han F, Ma Z, Chen C. 2025. Development and immunogenicity study of subunit vaccines based on spike proteins of porcine epidemic diarrhea virus and porcine transmissible gastroenteritis virus. Vet Sci 12:106. doi:10.3390/vetsci12020106 40005866 PMC11860644

[36] Du L, Tai W, Zhou Y, Jiang S. 2016. Vaccines for the prevention against the threat of MERS-CoV. Expert Rev Vaccines 15:1123–1134. doi:10.1586/14760584.2016.1167603 26985862 PMC5097835

[37] Meena J, Singhvi P, Srichandan S, Dandotiya J, Verma J, Singh M, Ahuja R, Panwar N, Wani TQ, Khatri R, Siddiqui G, Gupta A, Samal S, Panda AK. 2022. RBD decorated PLA nanoparticle admixture with aluminum hydroxide elicit robust and long lasting immune response against SARS-CoV-2. Eur J Pharm Biopharm 176:43–53. doi:10.1016/j.ejpb.2022.05.008 35589003 PMC9110063

[38] Shi Y, Shi J, Sun L, Tan Y, Wang G, Guo F, Hu G, Fu Y, Fu ZF, Xiao S, Peng G. 2021. Insight into vaccine development for Alpha-coronaviruses based on structural and immunological analyses of spike proteins. J Virol 95:e02284-20. doi:10.1128/JVI.02284-20 33414160 PMC8092709

[39] Song X, Li Y, Wang C, Zhao Y, Yang S, Guo R, Hu M, Sun M, Zhang G, Li Y, Wang Y, Liu S, Shen Y, Li C, Zhang X, Li J, Fan B, Li B. 2024. Efficacy evaluation of a bivalent subunit vaccine against epidemic PEDV heterologous strains with low cross-protection. J Virol 98:e0130924. doi:10.1128/jvi.01309-24 39254314 PMC11494954

[40] Guo W, Wang C, Song X, Xu H, Zhao S, Gu J, Zou Z, Li J, Qian J, Zhang X, Guo R, Li J, Li L, Hu Z, Ren L, Fan B, Li B. 2024. Immunogenicity and protective efficacy of a trimeric full-length S protein subunit vaccine for porcine epidemic diarrhea virus. Vaccine (Auckl) 42:828–839. doi:10.1016/j.vaccine.2024.01.020 38220489

[41] Yu M, Wang L, Ma S, Wang X, Wang Y, Xiao Y, Jiang Y, Qiao X, Tang L, Xu Y, Li Y. 2017. Immunogenicity of eGFP-marked recombinant Lactobacillus casei against transmissible gastroenteritis virus and porcine epidemic diarrhea virus. Viruses 9:274. doi:10.3390/v9100274 28946696 PMC5691626

[42] Hu GL, Luo X, Liao JM, Zou CC, Huang YH, Geng R, Zhao ZQ, Shen HQ, Cao YC, Peng O, Zhang H. 2025. Neutralizing antibody levels as a key factor in determining the immunogenic efficacy of the novel PEDV alpha coronavirus vaccine. Veterinary Quarterly 45:1–20. doi:10.1080/01652176.2025.2509506 PMC12120861 40432512

[43] Saha S, Raghava GPS. 2006. Prediction of continuous B-cell epitopes in an antigen using recurrent neural network. Proteins 65:40–48. doi:10.1002/prot.21078 16894596

[44] Grifoni A, Sidney J, Zhang Y, Scheuermann RH, Peters B, Sette A. 2020. A sequence homology and bioinformatic approach can predict candidate targets for immune responses to SARS-CoV-2. Cell Host Microbe 27:671–680. doi:10.1016/j.chom.2020.03.002 32183941 PMC7142693

[45] Saha S, Raghava GPS. 2004. BcePred: prediction of continuous B-cell epitopes in antigenic sequences using physico-chemical properties. Artificial immune systems. Third International Conference, ICARIS 2004; Catania, Sicily, Italy: , Vol. 3239, p 197–204

[46] Zhang D, Xie Y, Liao Q, Jiao Z, Liang R, Zhang J, Zhang Y, Tan Y, Wang H, Zhang W, Xiao S, Peng G, Shi Y. 2024. Development of a safe and broad-spectrum attenuated PEDV vaccine candidate by S2 subunit replacement. J Virol 98:e0042924. doi:10.1128/jvi.00429-24 39404450 PMC11575183

